# Development and validation of an MRI-radiomics nomogram for the prognosis of pancreatic ductal adenocarcinoma

**DOI:** 10.3389/fonc.2023.1074445

**Published:** 2023-02-24

**Authors:** Xinsen Xu, Jiaqi Qu, Yijue Zhang, Xiaohua Qian, Tao Chen, Yingbin Liu

**Affiliations:** ^1^ Department of Biliary-Pancreatic Surgery, Renji Hospital, School of Medicine, Shanghai Jiao Tong University, Shanghai, China; ^2^ School of Biomedical Engineering, Shanghai Jiao Tong University, Shanghai, China; ^3^ Department of Anesthesiology, Renji Hospital, School of Medicine, Shanghai Jiao Tong University, Shanghai, China; ^4^ Shanghai Key Laboratory of Biliary Tract Disease, Renji Hospital, School of Medicine, Shanghai Jiao Tong University, Shanghai, China; ^5^ Shanghai Research Center of Biliary Tract Disease, Renji Hospital, School of Medicine, Shanghai Jiao Tong University, Shanghai, China; ^6^ State Key Laboratory of Oncogenes and Related Genes, Shanghai Cancer Institute, Shanghai, China

**Keywords:** pancreatic cancer, radiomics, MRI, nomogram, prognosis

## Abstract

**Objective:**

To develop and validate an MRI-radiomics nomogram for the prognosis of pancreatic ductal adenocarcinoma (PDAC).

**Background:**

“Radiomics” enables the investigation of huge amounts of radiological features in parallel by extracting high-throughput imaging data. MRI provides better tissue contrast with no ionizing radiation for PDAC.

**Methods:**

There were 78 PDAC patients enrolled in this study. In total, there were 386 radiomics features extracted from MRI scan, which were screened by the least absolute shrinkage and selection operator algorithm to develop a risk score. Cox multivariate regression analysis was applied to develop the radiomics-based nomogram. The performance was assessed by discrimination and calibration.

**Results:**

The radiomics-based risk-score was significantly associated with PDAC overall survival (OS) (P < 0.05). With respect to survival prediction, integrating the risk score, clinical data and TNM information into the nomogram exhibited better performance than the TNM staging system, radiomics model and clinical model. In addition, the nomogram showed fine discrimination and calibration.

**Conclusions:**

The radiomics nomogram incorporating the radiomics data, clinical data and TNM information exhibited precise survival prediction for PDAC, which may help accelerate personalized precision treatment.

**Clinical trial registration:**

clinicaltrials.gov, identifier NCT05313854.

## Background

Pancreatic cancer, the 4^th^ lethal malignancies worldwide, is usually associated with late diagnosis and poor prognosis. The 5-year overall survival rate is less than 10% (1). With respect to pancreatic cancer types, nearly 95% are exocrine cancers and 5% are endocrine cancers (2). As the most common exocrine cancer, pancreatic ductal adenocarcinomas (PDAC) accounts for 90% of all pancreatic cancer (3). Compared with endocrine cancers, the degree of malignancy of exocrine cancers is generally higher and the prognosis is usually poorer. More than 80% of the PDAC patients are unresectable at the time of diagnosis, due to vessel invasion or distant metastasis (4). Thus, if poor survival can be identified at the time of diagnosis, aggressive treatments could be avoided for these patients.

Although the American Joint Committee on Cancer (AJCC) TNM staging system is the commonly used prognostic prediction system for pancreatic cancer, the prediction accuracy is not satisfied (5). Tremendous efforts have been made to explore the potential prognostic markers for pancreatic cancer. Previously, Liu et al. reported that serum CA199, CA125 and CEA levels were associated with patient survival. However, these markers are easily affected by obstructive jaundice in some pancreatic cancer patients (6). Collisson et al. reported that molecular profiling might also predict the prognosis for pancreatic cancer (7). Nevertheless, the clinical utility still needs to be verified.

The dual-phase enhanced CT scanning is recommended by the National Comprehensive Cancer Network (NCCN) guideline to assess the resectability and tumor stage for pancreatic cancer, mostly based on the tumor size and vessel invasion of the common hepatic artery or superior mesenteric artery (8). However, only based on these criteria, numerous other radiological quantitative features are ignored. Fortunately, “radiomics” enables the investigation of huge amounts of radiological features in parallel by extracting high-throughput imaging data (9). Recently, Xie et al. reported that the model based on radiomics data outperformed clinical model and TNM staging system for the prediction of PDAC prognosis (10).

Unlike CT, MRI depicts the tumor in more detail and reflects more physiologic characteristics. With respect to PDAC, MRI provides better tissue contrast with no ionizing radiation. However, to our knowledge, there is no study evaluating the predictive value of MRI radiomics signature for PDAC survival.

Therefore, we aim to explore the prognostic value of MRI-radiomics feature and develop and validate a nomogram incorporating both MRI-radiomics signature and clinical data for survival prediction for PDAC.

## Materials and methods

### Study population

This study (clinical-trial ID: NCT05313854-clinicaltrials.gov) was approved by the Ethical Committee of Renji Hospital, Shanghai Jiao Tong University. A total of 78 PDAC from 2014 to 2020 were enrolled in this study. The inclusion criteria were: 1) histologically confirmed PDAC; 2) with complete contrast-enhanced MRI data; 3) with complete TNM staging information and tumor markers data (CA199, CEA and CA125). The exclusion criteria were: 1) malignancy other than PDAC; 2) with missing survival information.

### MRI acquisition and image segmentation

The arterial phase of T1 sequences with contrast (TE 1.29-3.22ms, TR 3-6ms, slice thickness 3-6mm, FOV 80-100mm, flip angle 8.9-15.0°) were used, and the scan sequence is gradient recalled and research mode. MRIs of each patient were imported to the ITK-SNAP software (http://www.itksnap.org/). The three-dimensional segmentation of region of interest (ROI) was performed in all axial slices with 3 mm thickness by two experts of radiology (Xu X and Chen T) manually. The ROI covered the whole tumor and was delineated on contrast-enhanced T1-weighted images on each slice.

### Feature extraction and feature selection

The radiomics features of the ROI were extracted by PyRadiomics v3.0.1, which is an open-source python package (11). Pyradiomics can calculate various quantitative metrics from images using data-characterization algorithms based on morphology, intensity histogram and texture analyses. Here we calculated the features based on size and shape, descriptors of the image intensity histogram, and texture features from original images and wavelet-transformed images.

After feature extraction, the least absolute shrinkage and selection operator (LASSO) method was applied to identify the most relevant features to survival. LASSO is a kind of regression model which uses an L1 penalty to shrink some of the regression coefficients to zero during fitting. Therefore, a regression model between features and survival time was built by LASSO with the aim of minimizing the error between prediction value and true value to determine the optimal features. Then, a risk-score was calculated for each patient using a linear combination of the selected features and the weights computed by LASSO.

### Potential correlation of the risk-score and prognosis

To assess the potential association between the prognosis status and the risk-score, we classified the patients into two groups, namely, the high risk group and low risk group, based on the median value of the risk score. The Kaplan–Meier method was applied to plot the survival curve of each group. The log-rank test was utilized to assess the difference between the two groups to confirm the validity of the selected features.

### Development and assessment of the nomogram based on radiomics data

A multivariate Cox proportional-hazards model was built using the risk score and clinical features (age, sex, TNM staging information). Then a radiomics nomogram derived from this model was constructed to predict the prognosis at different scenarios. To evaluate the discriminant performance of the radiomics nomogram, Harrell’s concordance-index (C-index) was calculated. Besides, the calibration performance was qualified by the calibration curve, which described the consistency between the survival probability prediction and the observed value. To better verify the method of the proposed method, we used two verification methods (splitting dataset into 7:3 for train: test and four-fold cross-validation).

### Statistics

The statistical analysis was performed with Python programming language (version 3.6.8; https://www.python.org) and R software (version 4.0.4; http://www.R-project.org). The ‘sklearn’ package in python was used for executing the LASSO. The Cox-multivariate regression model, nomogram and calibration curve were completed using the ‘rms’ package in R software. The C-index was computed using the ‘lifelines’ package in python and the ‘Hmisc’ package in R software. Overall survival (OS) was defined as the length of time from the admission time to final observation (in days). 95% CI was applied to show the range of estimation for C-index.

## Results

### Patient characteristics

The baseline characteristics of the study patients were shown in **Table 1**. There were 48 male and 30 female patients. The mean age was 64 years old. The average tumor size was 3.89cm. According to the TNM staging system, there were 30 Stage I patients, 18 Stage II patients, 16 Stage III patients, and 14 Stage IV patients. The average level of CEA, CA199 and CA125 were 3.03 ng/ml, 94.2 U/ml, and 15.05 U/ml, respectively.

**Table 1:** Baseline characteristics of the study cohort.

Variables	
Ages (years)	64.2 ± 10.4
Sex (Male/Female)	48/30
Tumor size (cm)	3.89 ± 2.05
Tumor stage (I/II/III/IV)	30/18/16/14
T stage (1/2/3/4)	14/31/14/19
N stage (0/1/2)	43/25/10
M stage (0/1)	63/15
CEA (ng/ml)	3.03 (2.01 ~ 6.98)
CA199 (U/ml)	94.2 (31.4 ~ 371.2)
CA125 (U/ml)	15.05 (10.68 ~ 33.4)

### Risk-score building and evaluation

**Figure 1:**
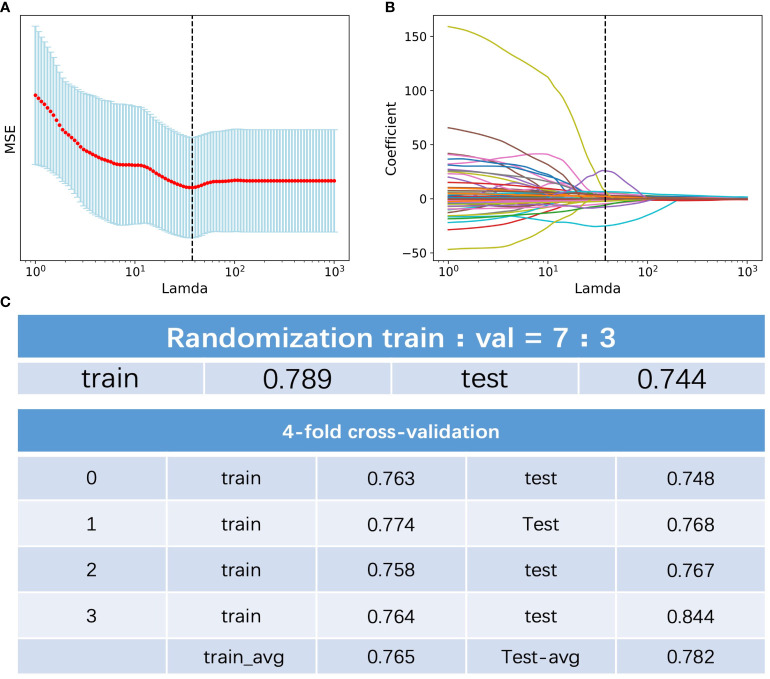
Feature selection of the radiomics data. **(A)** Selection of the tuning parameter lamda in the LASSO model. **(B)** LASSO coefficient profiles of the selected texture features. **(C)** C-index of the of the 11 features cox regression and validation by the randomization of training and testing cohorts.

**Figure 2:**
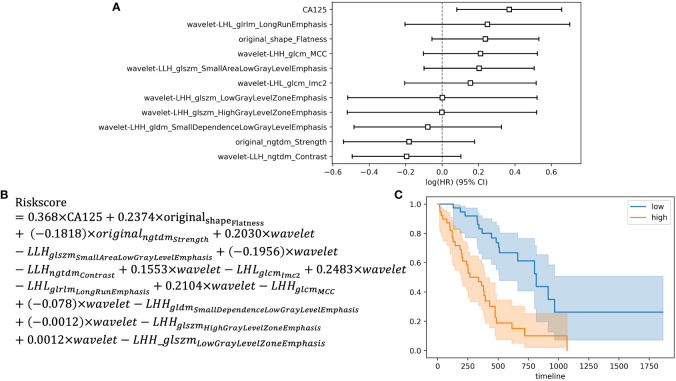
Development of the risk score and Kaplan-Meier survival analysis based on the risk score. **(A)** Forest plot showing hazard ratios for selected features. **(B)** Development of the risk score. **(C)** Kaplan-Meier survival curves for the patients in high risk group and low risk group.

Among the 396 features in total, which included 386 radiomics features (original features, wavelet-LHH, wavelet-LHL and wavelet-LLH) and 10 clinical features (tumor markers, age, sex, tumor size and TNM staging information), 11 were selected by LASSO regression (**Figures 1A, B**). The c-index of the 11 features cox regression was 0.75, which was validated by the randomization of training and testing cohorts (**Figure 1C**). Weighted by their respective coefficients in LASSO, the risk score was built (**Figures 2A, B**). The overall survival of the low-risk patients was significantly longer than that of the high-risk patients (**Figure 2C**).

### Development and assessment of the nomogram based on radiomics

**Figure 3:**
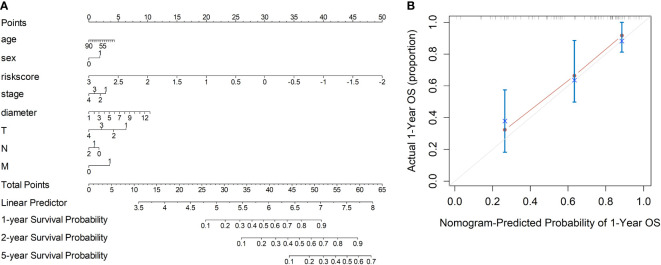
Development of the nomogram incorporating the radiomics data, clinical data and TNM information. **(A)** Development of the nomogram. **(B)** Calibration curves of the nomogram.

Based on the radiomics data, the nomogram that incorporated age, sex, TNM staging information and risk-score was constructed (**Figure 3A**). With respect to overall survival, the C-index of the radiomics nomogram was 0.78. In addition, fine agreement between observation and prediction was observed from the calibration curve plot (**Figure 3B**).

**Figure 4:**
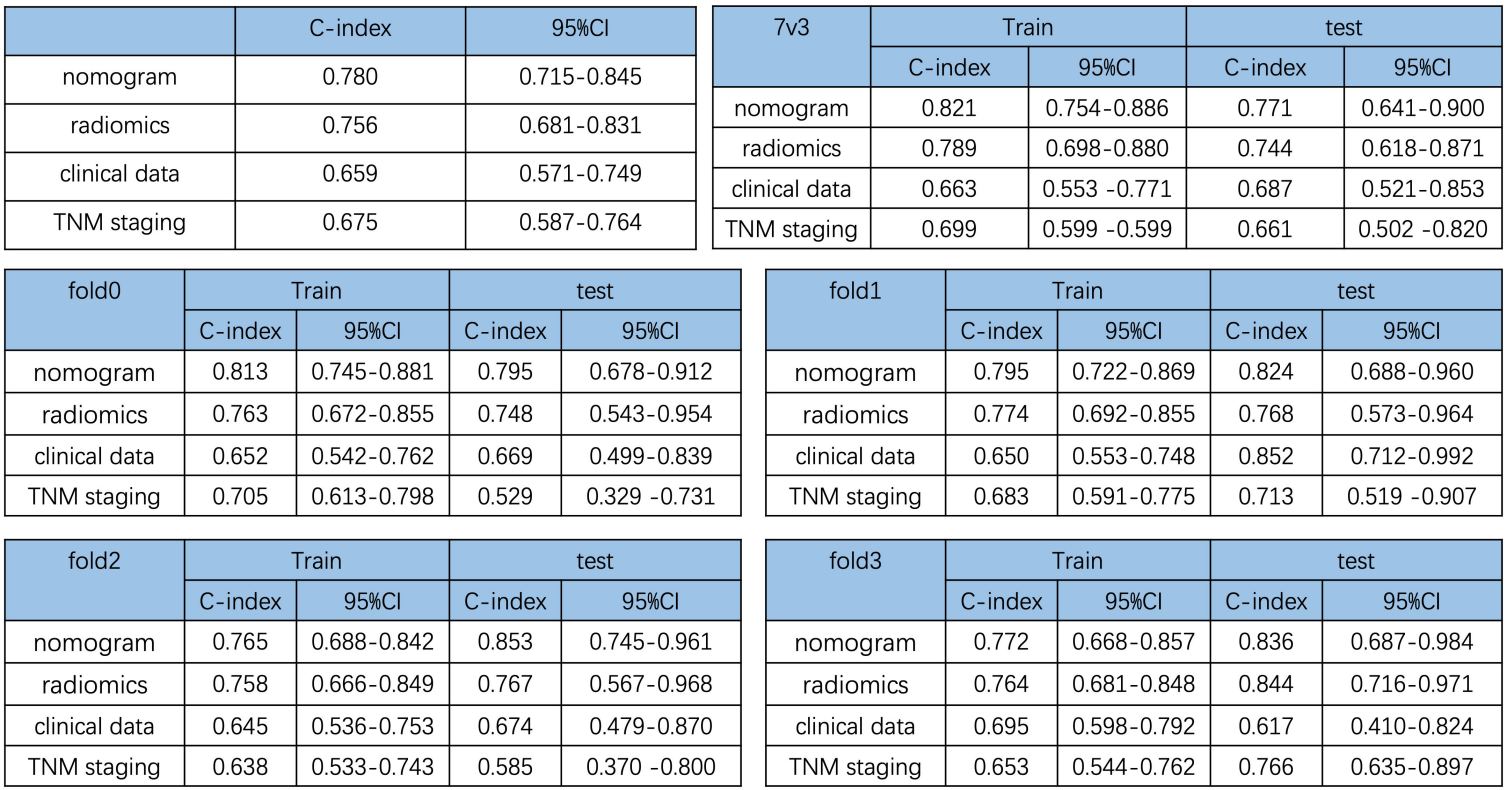
Comparison of the prediction ability of the nomogram and different cox-multivariate regression models. C-index of the different models and validation by the randomization of training and testing cohorts.

In addition, to show the prediction ability of the nomogram model, we also constructed three Cox-multivariate regression models based on solely radiomics data, clinical data, and TNM staging data, respectively. In comparison with each model, the combination nomogram exhibited the best prognostic discrimination performance with the highest c-index value in both the training and validation group (**Figure 4**).

## Discussion

In this study, we developed and validated a prognostic nomogram based on MRI radiomics signature for the survival prediction of PDAC. With respect to prediction accuracy, the highest C-index of the nomogram indicated that our model outperformed the traditional TNM staging system, clinical data model and the radiomics data model.

Accurate prediction of prognosis is crucial for the management of PDCA. However, with respect to the commonly utilized TNM staging system, which was mostly based on CT estimates of the tumor size and vessel invasion of the common hepatic artery or superior mesenteric artery, the prediction accuracy was not satisfied. This might be due to different biological features of tumors at the same stage. Thus, a new prognostic model which included both morphological features and biological features might exhibit better performance.

Fortunately, unlike CT, MRI depicts the tumor in more detail and reflects more physiologic characteristics. On the other hand, the radiomics extracts quantitative morphological features that are invisible to the human eye. Thus, the MRI-based radiomics data could provide more morphological and biological information. Our MRI-based radiomics signature successfully identified patients of high risk or low risk. Incorporating the radiomics signature and clinical data, TNM staging data into our nomogram improved the individualized survival prediction.

Previous studies have shown that radiomics features are associated with patient survival in different types of cancer (9, 12–17). In a recent study, Xie et al. reported that the radiomics nomogram based on CT scan data achieved a C-index of 0.742 for PDAC survival prediction. Compared with their results, our model achieved a C-index of 0.780, which outperformed the CT-based radiomics model. The limitations of our study include the relatively small sample size and lack of external validation. Studies of multi-center larger sample size are needed for future validation.

## Conclusion

In conclusion, our prognostic nomogram based on MRI radiomics signature outperformed the traditional TNM staging system, clinical data model and the radiomics data model for the survival prediction of PDAC.

## Data availability statement

The raw data supporting the conclusions of this article will be made available by the authors, without undue reservation.

## Ethics statement

The studies involving human participants were reviewed and approved by Shanghai Jiaotong University School of Medicine, Renji Hospital Ethics Committee. The patients/participants provided their written informed consent to participate in this study.

## Author contributions

XX: conceptualization, funding acquisition and drafted the work; JQ and YZ: data curation and statistical analysis; XQ: conceptualization and revision; TC and YL: conceptualization, review and editing. All authors contributed to the article and approved the submitted version.
